# *Myxococcus xanthus* Encapsulin as a Promising Platform for Intracellular Protein Delivery

**DOI:** 10.3390/ijms232415591

**Published:** 2022-12-09

**Authors:** Anna N. Gabashvili, Nelly S. Chmelyuk, Viktoria A. Sarkisova, Pavel A. Melnikov, Alevtina S. Semkina, Aleksey A. Nikitin, Maxim A. Abakumov

**Affiliations:** 1 Laboratory “Biomedical Nanomaterials”, National University of Science and Technology “MISiS”, Leninskiy Avenue, 4, 119049 Moscow, Russia; 2 Department of Medical Nanobiotechnology, Pirogov Russian National Research Medical University, Ostrovityanova Street, 1, 117997 Moscow, Russia; 3 Biology Faculty, Lomonosov Moscow State University, Leninskiy Gory, 119234 Moscow, Russia; 4 Cell Proliferation Laboratory, Engelhardt Institute of Molecular Biology, Vavilova Street, 32, 119991 Moscow, Russia; 5 Department of Basic and Applied Neurobiology, Serbsky National Medical Research Center for Psychiatry and Narcology, Kropotkinskiy Lane, 23, 119991 Moscow, Russia

**Keywords:** encapsulins, fluorescence, photoactivatable label, protein delivery

## Abstract

Introducing a new genetically encoded material containing a photoactivatable label as a model cargo protein, based on *Myxococcus xanthus* (*Mx*) encapsulin system stably expressed in human 293T cells. Encapsulin from *Mx* is known to be a protein-based container for a ferritin-like cargo in its shell which could be replaced with an exogenous cargo protein, resulting in a modified encapsulin system. We replaced *Mx* natural cargo with a foreign photoactivatable mCherry (PAmCherry) fluorescent protein and isolated encapsulins, containing PAmCherry, from 293T cells. Isolated *Mx* encapsulin shells containing photoactivatable label can be internalized by macrophages, wherein the PAmCherry fluorescent signal remains clearly visible. We believe that a genetically encoded nanocarrier system obtained in this study, can be used as a platform for controllable delivery of protein/peptide therapeutics in vitro.

## 1. Introduction

To date, therapies using various types of peptides are demonstrating their great potential in the treatment of diseases due to higher specificity, higher bioactivity and lower toxicity. Such proteins/peptides as insulin, growth factors, monoclonal antibodies and many others have been developed and applied in the clinical treatment of cancer, endocrinological diseases and other pathologies [1]. However, there are several difficulties associated with the use of such protein-based drugs due to their short lifetime and in vivo instability. Encapsulation of therapeutic peptides can prevent degradation and prolong circulation time.

There are a number of systems which can be used for encapsulation of therapeutic peptides/proteins such as: inorganic nanoparticles, liposomes, vesicles, etc [2]. For example, pluronics (in particular, PF127) have already been approved by FDA for insulin, interleukin-2 and various hormone delivery systems [3]. Polysaccharides such as chitosan and dextran were successfully applied for loading various protein/peptide therapeutics of IgG, VEGF (vascular endothelial growth factor) [4] and EGF (epidermal growth factor) [5]. Hydrogel-based systems for protein/peptide therapeutics delivery were also used. For example, collagen/hyaluronan, albumin/hyaluronan and gelatin incorporated hydrogels were designed to load EGF [6], Cx43MP (Connexin43 mimetic peptide) [7] and BMP-2 (bone morphogenetic protein) [8], respectively. Liposomes are the most studied nanocarrier systems [9] and they can be used for encapsulation of therapeutic proteins. The liposomes allowed containing granzyme B in a combination with mRNA [10]. There are other delivery systems for therapeutic proteins/enzymes; however, the goal of nanocarrier systems is to preserve the activity of loaded protein. 

Despite the plethora of tools available, the development of new protein/peptide delivery systems is an extremely urgent task. In this work, we present a genetically encoded nanocarrier based on *Myxococcus xanthus (Mx)* encapsulin system, containing photoactivatable mCherry (PAmCherry) fluorescent cargo protein label as a model cargo protein, expressed in 293T cells. Encapsulins are bacterial/archaeal high molecular weight capsid-like nanocompartments involved in various aspects of bacterial/archaeal metabolism [11], for example, wild-type *Mx* encapsulins are able to play a role in iron sequestration. *Mx* encapsulin consists of a protein shell (~32 nm diameter) which is self-assembled from 180 protomer proteins with a molecular weight equal to 32.5 kDa each, and ferritin-like cargo proteins (MxBCD). The protein shell of *Mx* encapsulin is encoded by the EncA gene [12]. The biological purpose of the cargo proteins encapsulation in bacteria and archaea is still unknown; however, some data support the hypothesis that encapsulation can increase the stability or lifetime of cargo proteins. It is also well known that encapsulin protein shells are extremely stable against pH and temperature changes [13,14,15] which is important for the preservation of cargo protein. 

Previously, the possibility of transient expression of encapsulins loaded with PAmCherry protein was shown in HEK293T cell line and it was shown that encapsulated PAmCherry protein retains its fluorescent properties [16]. In our work, a cell line stably expressing encapsulin system with PAmCherry protein as a model cargo was obtained by lentiviral transduction, which allows to produce encapsulins in 293T cells for a long period of time. Moreover, we have shown that isolated encapsulins from a stable cell line can be internalized by macrophages retaining PAmCherry fluorescent activity up to 2 h. Therefore, in this paper we present an extremely robust, genetically encoded nanocontainer system based on bacterial *Mx* encapsulins stably expressed in human 293T cells. Genes encoding *Mx* encapsulin shell protomer proteins (EncA-FLAG), and genes encoding photoactivatable mCherry protein were inserted into 293T cells genome via lentiviral transduction. This technique allows to obtain a stable cell line expressing encapsulated photoactivatable mCherry label (293T EncA_PAmCherry cells). Encapsulated PAmCherry label can be isolated from 293T EncA_PAmCherry cells by immunoprecipitation. Isolated encapsulin shells containing photoactivatable cargo protein can be successfully internalized by RAW 264.7 cells (mouse macrophage-like cell line) wherein the PAmCherry fluorescent signal remains clearly distinguishable, proving that the loaded PamCherry protein remains intact and functionally active. In light of the aforementioned, we can assume that the genetically encoded nanocarrier obtained in this study can be used in the future as a possible platform for nanoshell-based protein delivery tools. 

## 2. Results

### 2.1. Expression of Encapsulated PAmCherry in 293T Cells

**Figure 1:**
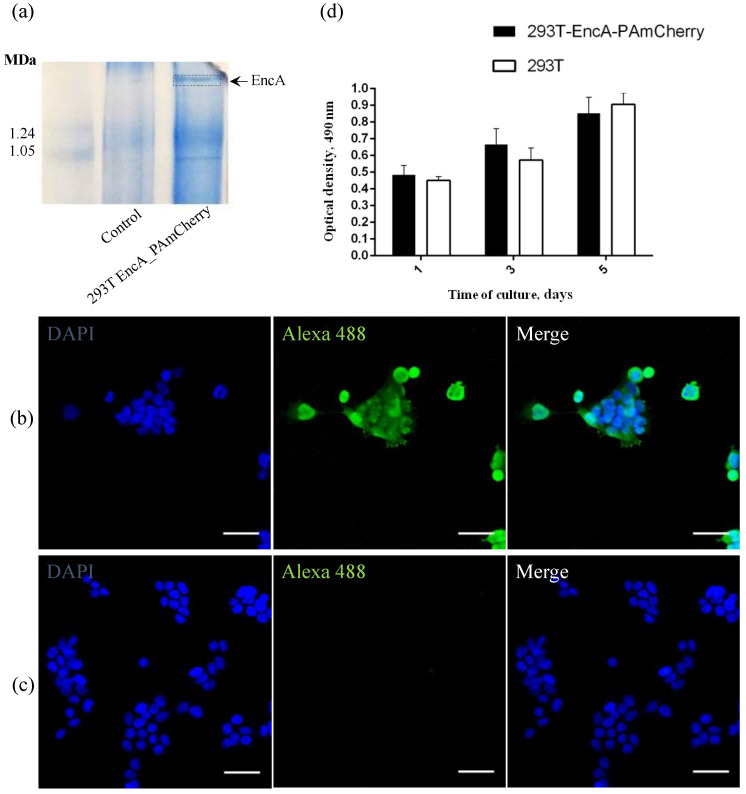
(**a**) Coomasie-stained BN-PAGE gels loaded with 293T and 293T EncA_PAmCherry cell lysates. Arrow and dotted line indicate the band corresponding to *Mx* EncA protein shells; (**b**) 293T EncA_PAmCherry and (**c**) 293T cells stained with Alexa Fluor 488 anti-DYKDDDDK Tag antibody (green fluorescence signal). Nuclei were counterstained with DAPI (blue fluorescence signal). Laser scanning confocal microscopy, Nikon Eclipse Ti2, scale bars are 50 μm; (**d**) Influence of the presence of a genetically encoded label on viability and proliferation of 293T EncA_PAmCherry cells. Optical density is proportional to the number of living cells. The numbers of living cells were not significantly different in 293T EncA_PAmCherry and 293T cells. The data are shown as the mean + S.D. of three independent experiments, *p* values were calculated using a one-tailed *t*-test, assuming unequal variances.

To achieve stable expression of PAmCherry fluorescent protein encapsulated into *Mx* encapsulin shell in 293T cells, we performed lentiviral transduction. We used two viral vectors: the first one carried *Mx* encapsulin protomer protein encoding genes (EncA, tagged with a FLAG sequence), and the second one carried genes encoding fluorescent PAmCherry cargo. As previously mentioned, the PAmCherry encoding gene is coupled to a destabilization domain, which leads to degradation of the PAmCherry protein if not encapsulated into *Mx* shell. Blue Native PAGE (BN-PAGE) analysis of 293T EncA_PAmCherry cells is presented in Figure 1a which shows one band corresponding to assembled *Mx* encapsulin shells and it is clearly seen that such a band is absent in the control (non-transduced) 293T cells.

To detect the FLAG sequence co-expressed with EncA on encapsulin protomer proteins in 293T EncA_PAmCherry cells, we also performed direct immunostaining using monoclonal anti-DYKDDDDK Tag monoclonal antibodies labeled with Alexa Fluor 488 label (Ex-Max 495 nm/Em-Max 519 nm) and 293T cells were used as a control. The high intensity green fluorescent signal from Alexa Fluor 488 label in 293T EncA_PAmCherry cells (Figure 1b) is visible in the micrograph. Fluorescent signal wasn’t detected in non-transduced 293T cells stained with anti-DYKDDDDK Tag antibodies (Figure 1c).

To quantitatively evaluate whether the presence of a genetically encoded label affects 293T EncA_PAmCherry cells viability and proliferation rates, we compared the dynamics of growth of intact 293T and 293T EncA_PAmCherry cells on different days (1, 3, 5) of culture using MTS assay in which the absorbance at 490 nm is proportional to the number of living cells. The proliferation rates of 293T EncA_PAmCherry and intact 293T cells (Figure 1d) are not statistically different. Therefore, we can conclude that there are no negative effects on cell viability and proliferation associated with the presence of a genetically encoded label in 293T EncA_PAmCherry cells.

### 2.2. Encapsulated PAmCherry Photoactivation in Cells

**Figure 2:**
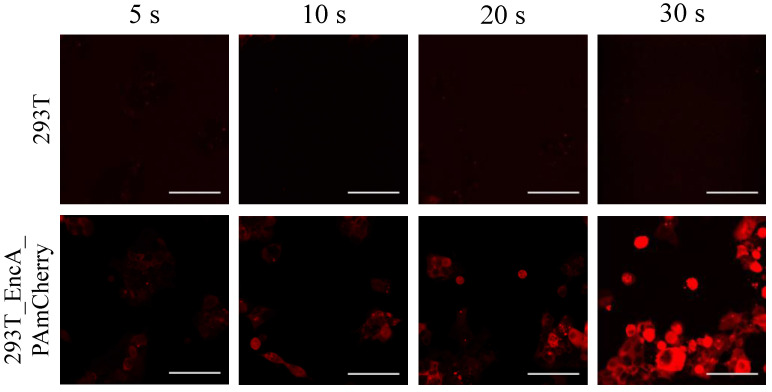
293T (**upper panel**) and 293T EncA_PAmCherry (**lower panel**) cells were imaged after irradiation with 405 nm laser for 5 s, 10 s, 20 s and 30 s followed by 561 nm excitation. Laser scanning confocal microscopy, Nikon Eclipse Ti2, scale bars are 50 μm.

We performed confocal microscopy photoactivation experiments in 293T EncA_PAmCherry cells using intact 293T cell as a control. Initially (before photoactivation) no fluorescence signal in the red channel was detected; however, after irradiation with blue light followed by 561 nm excitation, the intensity of red fluorescence signal in 293T EncA_PAmCherry cells increased dramatically (Supplementary Video S3). Following photoactivation of EncA_PAmCherry with 405 nm laser light over various periods of time, fluorescence signal in the cytoplasm was detected, which progressively increased over the course of 30 s (Figure 2, lower panel). Supplementary Video S1 demonstrates life imaging of the photoactivation process in 293T EncA_PAmCherry cells during simultaneous irradiation with 405 nm and 561 nm lasers.

In contrast, fluorescence signal wasn’t detected in 293T cells neither before nor after photoactivation (Figure 2, upper panel; Supplementary Video S2).

### 2.3. Uptake of Encapsulated Fluorescent Cargo Protein by RAW 264.7 Cells

**Figure 3:**
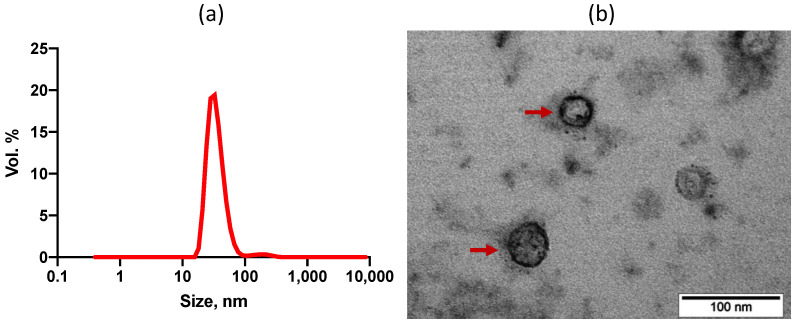
(**a**) Dynamic light scattering analysis of EncA_PAmCherry (34 ± 4 nm; PDI 0,22). Hydrodynamic size of *Mx* encapsulins, volume distribution. (**b**) Bright-field TEM image of Uranyless -stained EncA_PAmCherry, red arrows indicate *Mx* encapsulins protein shells, scale bar 100 nm.

To further study the fate of encapsulated PAmCherry cargo in cells, we isolated encapsulins from 293T EncA_PAmCherry cells using the immunoprecipitation method. Hydrodynamic size (Figure 3a) of isolated encapsulins (EncA_PAmCherry) in TBS buffer measured by dynamic light scattering method amounted to 34 ± 4 nm with 0,22 PDI (polydispersity index). We also performed TEM imaging of isolated EncA_PAmCherry (Figure 3b). 

**Figure 4:**
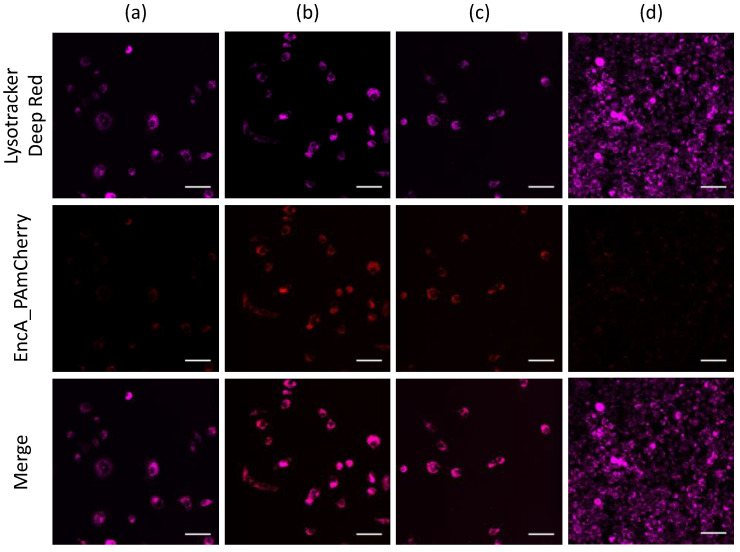
Confocal imaging of RAW 264.7 cells after (**a**) 15 min, (**b**) 1 h, (**c**) 2 h and (**d**) 24 h of incubation with isolated EncA_PAmCherry. RAW 264.7 cells were imaged after irradiation with 405 nm laser for 30 s followed by 561 nm excitation of PAmCherry label (red fluorescence signal). Lysosomes were stained with LysoTracker Deep Red dye (purple fluorescence signal). Laser scanning confocal microscopy, Nikon Eclipse Ti2 and scale bars are 50 μm.

Isolated encapsulins containing PAmCherry label were added to RAW 264.7 cells. After the addition of EncA_PAmCherry to the macrophage cells and subsequent incubation for 15 min, 1 h, 2 h and 24 h, lysosomes were additionally stained with LysoTracker Deep Red (Ex-Max 647 nm/Em-Max 668 nm) and confocal microscopy imaging of the cells was performed as described above. It was found (Figure 4a) that after 15 min of incubation, PAmCherry fluorescence signal in the red channel was already quite low but clearly visible. Then, the intensity of the signal gradually increased with an increase in incubation time to 1 h (Figure 4b). The signal was still clearly distinguishable after 2 h of incubation of RAW 264.7 cells with EncA_PAmCherry (Figure 4c). Finally, after 24 h of incubation only minor fluorescence signal in the red channel was detected (Figure 4d). We assume that *Mx* protein shells and PAmCherry label were digested by the cells.

Notably, the part of the PAmCherry signal was colocalized with LysoTracker far-red fluorescence signal. Further experiments are needed to determine the mechanisms of *Mx* encapsulin nanoshell endocytosis. 

## 3. Discussion

In a number of previously published articles, we have shown that stable heterologous expression of bacterial encapsulin shell encoding genes can be successfully achieved in mammalian cell lines, wherein the presence of heterogenous sequences in cells did not affect the rate of cell proliferation. One of our previous studies has shown that stable heterologous expression of *Mx* encapsulin genes can be achieved in human mesenchymal stem cells (MSCs). In that study, a lentiviral construct encoding the MxEncA-FLAG subsequence was used in conjunction with a lentiviral vector encoding the natural *Mx* BCD cargo protein and showed its high efficiency in MSCs transduction. [17]. We have also already obtained a mouse carcinoma cell line stably expressing *Quasibacillus thermotolerans* (*Qt*) encapsulin genes which allows the tracking of carcinoma cells via MRI [18]. Thus, our previous research has focused on the study of stable expressed *Mx* and *Qt* encapsulins in mammalian cells as genetically encoded reporters that enable non-invasive cell tracking. In the present study, the obtained cell line performs an auxiliary function allowing the production and isolation of *Mx* encapsulins. 

Current observations confirmed that encapsulins are able to encapsulate various types of engineered cargo proteins, for example TFP (teal fluorescent protein) [19], EGFP (enhanced green fluorescent protein), luc (firefly luciferase) [20], peroxidase [14] and many others. This feature allows for the use of encapsulin-based systems in a variety of applications such as reporter systems, nanoreactors, targeted drug delivery platforms and nanocarriers for biomaterials synthesis.

There is existing research describing the use of encapsulins as a nanocontainer system to deliver various types of cargo to cells. For example, in one study [21] *Thermotoga maritima* encapsulins were used as a system for targeted delivery of a therapeutic drug into HepG2 (hepatocellular carcinoma) cells. The shells of nanocompartments were modified by SP94 peptide which specifically binds to the GRP78 protein (Glucose-regulated protein 78 kDa), which is overexpressed in various tumor cells including HepG2 cell line. In another study there was found that TFP loaded encapsulins from *Brevibacterium linens* were successfully internalized by J774 mouse macrophages in vitro. The authors of this work noted that it is possible to replace TFP cargo protein with a therapeutic drug and use encapsulin-based technology for its delivery. Interestingly, in the above article, the internalization time of encapsulins into macrophages and, especially, the intracellular distribution of the fluorescent cargo signal differ significantly from what we describe. 

One of the latest works also describes *Thermotoga maritima*, *Mx* and *Qt* encapsulins as novel platforms for simultaneous RNA and protein packaging for their future use as targeted co-delivery systems. This allows for the targeting of multiple intracellular target classes (for example mRNA and protein, at the same time) [22]. 

Previously [16], PAmCherry protein has already shown itself as an optical label that can be encapsulated into *Mx* encapsulin shells. It was shown that photoactivation of PAmCherry protein inside the encapsulins could be detected by confocal microscopy—fluorescence signal was detected throughout the cytoplasm of HEK293T cells, which is in good agreement with our data. Additionally, in this study a non-integrative adeno-associated viral vector was used to deliver MxA-FLAG together with ferritin-like B^M7^ cargo into murine brains, allowing only transient gene expression to be achieved. Such an approach does not allow to obtain a cell line, stably expressing an encapsulin system. 

In the case when a cell line is used to produce encapsulins, the use of lentiviral vectors seems to be a more appropriate technique, since it allows to obtain a stable cell line once and then using it for a long period of time. Two lentiviral vectors were used to obtain a cell line stably expressing MxEncA encapsulin genes and PAmCherry encoding genes, which makes it possible to produce *Mx* encapsulins containing PAmCherry label in mammalian cells without the use of any bacterial strains. In our opinion, this is an important alternative strategy for the synthesis of encapsulins since the isolation of encapsulins from bacteria may be associated with bacterial endotoxin contamination. 

Confocal microscopy was performed to study the process of photoactivation of the fluorescent cargo protein in cells. After irradiation with 405 nm light followed by 561 nm excitation, the intensity of red fluorescence signal in 293T EncA_PAmCherry cells significantly increased. This observation is in agreement with the data obtained by Verkhusha and coauthors [15]. We can conclude, therefore, that encapsulation does not affect the process of PAmCherry photoactivation. 

To further demonstrate the functionality of PAmCherry containing *Mx* encapsulins for in vitro application, we isolated *Mx* encapsulins containing PAmCherry label from 293T EncA_PAmCherry cells by immunoprecipitation method. It was found that EncA_PAmCherry hydrodynamic size measured by DLS method was 34 ± 4 nm (0.22 PDI), which also agrees well with the TEM analysis data.

Laser scanning confocal microscopy was used to evaluate the uptake and intracellular distribution of EncA_PAmCherry by RAW 264.7 cells (macrophage-like, Abelson leukemia virus transformed cells). This cell line is often used to study the processes of internalization of various nanoparticles, including liposomes [23], gold nanoparticles [24], micelles [25] and many others. There was found that after 15 min of incubation, EncA_PAmCherry were already efficiently internalized by macrophages: fluorescent PAmCherry signal in the red channel which became demonstrable in the RAW 264.7 cells cytoplasm. The signal was still distinguishable after 2 h of incubation and the most of the PAmCherry fluorescence was colocalized with LysoTracker Deep Red fluorescence signal. After 24 h of incubation, only minor fluorescent signal in the red channel was detected, probably due to *Mx* protein shells and PAmCherry label digestion by the cells. Therefore, in this study we describe the possibility of intracellular delivery of functionally active protein inside of encapsulin shells into macrophage lysosomes. 

Further experiments are needed to determine the mechanism of *Mx* encapsulin endocytosis; however, we believe that our research opens an opportunity to use an encapsulin based system for intracellular protein delivery For example, encapsulin shells can be functionalized with specific ligands or monoclonal antibodies and the model PAmCherry cargo protein can be replaced with therapeutic protein/peptide to be applied as a targeted drug delivery system.

## 4. Materials and Methods

### 4.1. Cell Culture

293T cells were cultured in the CO_2_ incubator (Sanyo, Osaka, Japan), at 37 °C and 5% CO_2_ in DMEM culture medium supplemented with 10% FBS, 2 mM L-glutamine, 100 U/mL penicillin and 0.1 mg/mL streptomycin in T-25 flasks. Upon reaching 70–80% confluency, the cells were harvested by trypsinization and sub-cultured at a 1:3–1:8 ratio. 

RAW264.7 (macrophage-like, Abelson leukemia virus-transformed cell line derived from mice) cells were cultured in RPMI medium supplemented with 10% FBS, 100 U/mL penicillin, 0.1 mg/mL streptomycin and 2 mM L-glutamine in atmosphere of 5% CO_2_ and 80% humidity at 37 °C. RAW264.7 cells were passaged after reaching 80–90% confluence, detached with cell scraper and sub-cultivated in 1:4–1:6 ratio in T-25 flasks. Culture plastic was purchased from Corning (New York, NY, USA), all reagents for cell culture were acquired from Gibco (New York, NY, USA).

### 4.2. Construction of Lentiviral Particles and Lentiviral Transduction 

pRSV-Rev (19% by mass of total DNA), pMDLg/pRRE (37% by mass of total DNA), pCMV-VSV-G (7% by mass of total DNA) lentiviral packaging plasmids were used (all from Addgene (Watertown, MA, USA)). We also used one plasmid carrying encapsulin genes pCMV MxEncA-FLAG (37% by mass of total DNA) and another plasmid carrying genes of fluorescent cargo with the *Mx* encapsulation signal pLCMV DD-N_PAmCherry1_MxSig (37% by mass of total DNA) also used. Plasmids and P3000 reagent were mixed in Opti-MEM medium, then lipofectamine in Opti-MEM was added to the solution (1:1). The mixture was stirred and incubated for 20 min (room temperature) and then added dropwise to 293T cells. After 24 h of incubation, the initial growth medium was replaced with DMEM supplemented with 2 mM L-glutamine and 2% FBS. The supernatant containing viral particles was collected 48 h and 72 h post-transfection, loaded onto a 20 mL syringe and filtered through a 0.45 µm syringe filter (Merck, Rahway, NJ, USA). Transduction of the cells with the lentiviral vectors was performed according to standard protocol in DMEM growth medium supplemented with 10% heat-inactivated FBS and 8 μg/mL polybrene (Sigma-Aldrich, Burlington, MA, USA). Lentiviruses were added to give a multiplicity of infection of four for each virus, 48 h after transduction, the selection was started using puromycin (Thermo Fisher Scientific, Waltham, MA, USA) at a concentration of 2.5 µg/mL.

### 4.3. Blue Native Gel Electrophoresis

For detection of *Mx* encapsulin shell proteins, NativePAGE Novex 3–12% Bis-Tris gels (Life Technologies, Carlsbad, CA, USA) were used according to the manufacturer’s recommendations. Gels were loaded with whole cell lysates mixed with NativePAGE Novex sample buffer and run for 120 min at 150 V. Protein standard (Life Technologies, Carlsbad, CA, USA) covering a size range between 20 and 1200 kDa was used as a marker. Gels were then stained using Coomassie stain according to the manufacturer’s protocol (Bio-Rad Laboratories, Hercules, CA, USA). 

### 4.4. Immunofluorescence 

For immunofluorescence assay 293T and 293T EncA_PAmCherry cells were seeded on 3.5 cm^2^ µ-Dish with a polymer coverslip bottom for confocal microscopy (Ibidi, Martinsried, Bayern, Germany), in the amount of 3 × 10^4^ cells/dish, cultured for 24 h in standard conditions, fixed by 4% formaldehyde (Thermo Scientific, Waltham, MA, USA) in DPBS (Thermo Fisher Scientific, Waltham, MA, USA) and stained by anti DYKDDDDK Tag Alexa Fluor 488 Monoclonal Antibody (BioLegend, San Diego, CA, USA, 1:500), according to the manufacturer’s instructions. Cell nuclei were counterstained with DAPI (Sigma Aldrich, Burlington, MA, USA, 1:1000).

### 4.5. Laser Scanning Confocal Microscopy

Cell imaging was performed on either a Nikon Eclipse Ti2 (Nikon, Tokyo, Japan) with 405, 561 and 642 lasers (ThorLabs, Newton, NJ, USA), Apo 25X/1,1 water immersion objective lenses (Nikon, Tokyo, Japan) or on a Nikon A1R MP+ (405 and 561 lasers, Apo TIRF 60X/1.49 oil immersion objective lenses) laser scanning confocal microscope (Nikon, Tokyo, Japan). Scanning was performed using the ThorImageLS (version 2.4) Software (Thorlabs, Newton, NJ, USA) and Nikon NIS elements (version 4.50) software (Nikon, Tokyo, Japan), ImageJ2 Fiji (https://imagej.nih.gov/ij/, accessed on 1 November 2022) was used to process the images.

### 4.6. MTS Cell Proliferation Assay

For comparative assessment of the proliferation rate, 293T and 293T EncA_PAmCherry cells were seeded into 96-well plates in 100 uL of growth medium (5 × 10^4^ cells/well). After 1, 3 and 5 days of culturing 20 µL of MTS solution (Promega, Madison, WI, USA) was added to 100 µL of cell culture medium into each well. Then the cells were incubated with MTS reagent for 4 h and the optical density was measured using a Multiscan GO plate reader (Thermo Scientific, Waltham, MA, USA), λ = 490 nm.

### 4.7. Immunoprecipitation

293T EncA_PAmCherry cells were seeded into 6-well plates in 2 mL of growth medium (1.2 × 10^6^ cells/well). After 24 h cultivation FLAG-tagged encapsulins were isolated from 293T EncA_PAmCherry cells using Anti-DYKDDDDK Tag (L5) Affinity Gel (Sigma-Aldrich, Burlington, MA, USA) according to the manufacturer instructions. The cells were washed with DPBS and incubated with MPER cell lysis buffer (460 uL of buffer were added into each well) for 15 min on a shaker at 4 °C. Cell lysates were centrifugated at 10,000× *g* for 20 min at 4 °C, and the supernatant was incubated with pre-equilibrated gel for 1.5 h on a shaker at 4 °C. For elution, the gel was incubated with 100 μg/mL 3X FLAG Peptide (Sigma-Aldrich, Burlington, MA, USA) for 30 min at 4 °C and centrifugated at 5000× *g* for 1 min. The eluate was kept at 4 °C for further analysis.

### 4.8. Cellular Uptake of Isolated Encapsulins

RAW264.7 cells were seeded on 3.5 cm^2^ µ-Dish with a polymer coverslip bottom for confocal microscopy, in the amount of 1 × 10^5^ cells/dish and cultured for 24 h in standard conditions. Following cultivation, 60 μL of eluated *Mx* encapsulins was added to the cells. The cells were incubated at 37 °C and 5% CO_2_ for 15 min, 30 min, 1 h and 2 h to allow the cells to uptake the protein shells. Additionally, LysoTracker Deep Red (Thermo Fisher Scientific, Waltham, MA, USA) dye was added to a final concentration of 20 nM. Then, the medium was removed, and the cells were rinsed twice with DPBS. The cells were imaged using a Nikon Eclipse Ti2 confocal microscope (Nikon, Tokyo, Japan).

### 4.9. Dynamic Light Scattering

Eluate was diluted in TBS 1:3 rate and added in a glass cuvette. Hydrodynamic size obtained encapsulins were measured by ZetaSizer Nano ZS (Malvern Panalytical, UK), Zetasizer software. Measurements were performed at 25 °C using standard rectangular glass cuvettes containing 1000 μL of protein solution.

### 4.10. Transmission Electron Microscopy (TEM)

A suspension of encapsulins in TBS was dropped onto the surface of a formvar-coated copper grid (300 mesh), and the solvent was subsequently evaporated. Then encapsulins were incubated with UranyLess (Electron Microscopy Sciences, Hatfield, PA, USA) for 20 s, and after that the copper grid with encapsulins was rinsed twice in DI water. TEM analysis was performed on a JEM-1400 microscope (JEOL, Tokyo, Japan).

## 5. Conclusions

In summary, in this study we describe a new genetically encoded nanocarrier system based on bacterial *Mx* encapsulins stably expressed in human 293T cells. The container confines PAmCherry fluorescent cargo protein label which is used in this study as a model cargo protein. The presence of EncA-FLAG and PAmCherry sequences do not alter the proliferation of 293T EncA_PAmCherry cells. Genetically encoded nanocontainers obtained in this study can be easily isolated from 293T EncA_PAmCherry cells by immunoprecipitation, avoiding the use of any bacterial strains for the production of encapsulins. Isolated encapsulins containing PAmCherry cargo are able to internalize by RAW 264.7 cells while maintaining the fluorescent signal for at least 2 h, which allows us to assert that the protein shell protects the cargo protein. In the future, nanoshell-based genetically encoded nanomaterials obtained in this study can be vectorized with specific ligands such as transferrin whose receptor is overexpressed in tumor cells. The model cargo protein can be replaced with a therapeutic protein; therefore, an encapsulin-based vehicle can be applied as a targeted delivery system for cancer therapy.

## Data Availability

Not applicable.

## Supplementary Materials

The following supporting information can be downloaded at: https://www.mdpi.com/article/10.3390/ijms232415591/s1, Video S1: Photoactivation in 293T EncA_PAmCherry cells; Video S2: Photoactivation in 293T cells; Video S3: Photoactivation in 293T EncA_PAmCherry cells.
