# Supplementary material for: Myxococcus xanthus Encapsulin as a Promising Platform for Intracellular Protein Delivery
Source: Int J Mol Sci. 2022 Dec 9;23(24):15591. doi: 10.3390/ijms232415591 (PMC9778880; doi:10.3390/ijms232415591)
Supplement: Supplementary file 1 [file ijms-23-15591-s001.zip › description.pdf]

Video_S1 Photoactivation of encapsulated PAmCherry label in 293T EncA_PAmCherry cells during simultaneous irradiation with 405 nm and 561 nm lasers.

Video_S2 No photoactivation occurs in control (non-transduced) 293T cells. Plot of fluorescence intensity (Y axis, a.u.) vs. time (X axis, s)

Video_S3 Photoactivation of encapsulated PAmCherry label in 293T EncA_PAmCherry cells during simultaneous irradiation with 405 nm and 561 nm lasers. Plot of fluorescence intensity (Y axis, a.u.) vs. time (X axis, s).
